# BEATVIC, a body-oriented resilience therapy using kickboxing exercises for people with a psychotic disorder: a feasibility study

**DOI:** 10.1186/s12888-018-1958-6

**Published:** 2018-12-11

**Authors:** Bertine de Vries, Elisabeth C. D. van der Stouwe, Clement O. Waarheid, Stefan H. J. Poel, Erwin M. van der Helm, André Aleman, Johan Arends, Gerdina H. M. Pijnenborg, Jooske T. van Busschbach

**Affiliations:** 1 Department of clinical psychology and experimental psychopathology, faculty of behavioral and social sciences, University of Groningen, Grote Kruisstraat 2/1, 9712 TS Groningen, Netherlands; 2 University of Groningen, University Medical Center Groningen, University Center of Psychiatry, Rob Giel Onderzoekcentrum, Hanzeplein 1, 9713 GZ Groningen, Netherlands; 3 Department of Neuroscience, BCN Neuroimaging Center, University of Groningen, University Medical Center Groningen, Antonius Deusinglaan 2, 9713 AW Groningen, Netherlands; 4 Department of Psychotic Disorders, GGZ-Drenthe, Dennenweg 9, 9404 LA Assen, Netherlands; 5 Helmsport, Vechtstraat 72B, 9725 CW Groningen, Netherlands; 6 Department of Human Movement and Education, Windesheim University of Applied Sciences, Campus 2-6, 8017 CA Zwolle, the Netherlands

**Keywords:** Psychotic disorder, Psychomotor, Nonverbal therapy, Kickboxing, Victimization, Assertiveness, Social cognition, Self-esteem

## Abstract

**Background:**

People with a psychotic disorder have an increased risk of becoming the victim of a crime. To prevent victimization a body-oriented resilience therapy using kickboxing exercises was developed. This study aims to explore the feasibility of the therapy, to improve the therapy protocol and to explore suitable outcomes for a RCT.

**Methods:**

Twenty-four adults with a psychotic disorder received 20 weekly group sessions in which potential risk factors for victimization and strategies for dealing with them were addressed. Sessions were evaluated weekly. During pre and post assessment participants completed questionnaires on, among other, victimization, aggression regulation and social functioning.

**Results:**

The short recruitment period indicates the interest in such an intervention and the willingness of clients to participate. Mean attendance was 85.3 and 88% of the participants completed fifteen or more sessions. The therapy protocol was assessed as adequate and exercises as relevant with some small improvements to be made. The victimization and aggression regulation questionnaires were found to be suitable outcome measurements for a subsequent RCT.

**Conclusion:**

The results support the feasibility of the BEATVIC therapy. Participants subjectively evaluated the intervention as helpful in their attempt to gain more self-esteem and assertiveness. With some minor changes in the protocol the effects of BEATVIC can be tested in a RCT.

**Trial registration:**

The trial registration number (TRN) is 35949 (date submitted 09/11/2018). Retrospectively registered.

## Background

With psychotic disorder having a median global prevalence of 4.6 per 1000 persons [1], and this leading to a four to six times higher risk of becoming a victim of a crime [2, 3], the prevention of victimization in these already vulnerable people is an important public health concern [4]. However, currently there is no evidence-based intervention which aims to decrease the risk of victimization for people with a psychotic disorder.

To prevent victimization of people with a psychotic disorder, a body-oriented resilience therapy with kickboxing exercises was developed, henceforward referred to as BEATVIC [5]. This therapy is based on principles of what is called body-oriented psychotherapy in Anglo Saxon countries [6], or what in European countries is referred to as psychomotor therapy (PMT) [7]. PMT is an experience-based approach, which combines physical activity with body and emotional awareness [8].

**Fig. 1:**
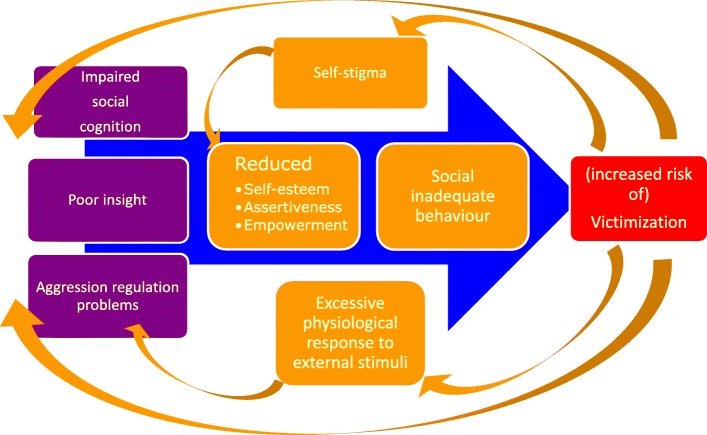
Proposed model of victimization risk factors

The intervention addresses several important risk factors that are assumed to be associated with victimization in individuals with a psychotic disorder, and which are amenable to change (see Fig. 1). First of all, social cognitive impairments are common in people with a psychotic disorder and may lead to difficulties in social functioning [9, 10] which is associated with victimization [11]. Another potential risk factor is poor insight. A lack of clinical and/or cognitive insight is associated with aggressive behaviour [12], which itself could elicit aggression in others [13], leading indirectly to victimization. Accordingly, another factor that is addressed in BEATVIC concerns problems in aggression regulation. Self-stigma, e.g. as a result of earlier victimization [14] could result in low self-efficacy [15], low self-esteem and reduced empowerment [16]. Consequently, people may experience difficulties standing up for themselves in social situations which makes them more prone to become victimized [17]. For people with psychosis, as for anyone else, the traumatic experience of being a victim may lead to hyper arousal including an increased physiological arousal [18] and emotion dysregulation. This could impair the ability to adequately detect or respond to risks and for this reason it may be associated with revictimization [19]. Victimized people often get revictimized, suggesting a vicious cycle, which is included in the model as well. For a more comprehensive explanation of risk factors see an earlier published paper [5].

A suitable intervention should address several of the suggested risk factors and encompass ways to deal with the underlying deficits and inadequate responses. From this perspective BEATVIC was developed. In this psychomotor intervention, positive effects of physical exercise (e.g. improve physical and psychological functioning) [20, 21], were combined with those of assertiveness training (e.g. increase self-esteem, assertiveness) [22, 23] and martial arts (e.g. positive effect on aggression regulation, empowerment and social interactions) [24–26]. To provide an activating, challenging and possibly destigmatizing context kickboxing was used as the basic form of exercise.

The current feasibility study was set up in preparation for a multicentre randomized controlled trial (RCT), aimed at investigating the effectiveness of BEATVIC. The aim of the current study was threefold: (1) to explore the feasibility of the intervention and application of a RCT; (2) to improve the intervention protocol; (3) to explore suitable outcome measures for a possible subsequent RCT.

## Methods

This feasibility study had a pretest-posttest quasi-experimental design without a control group.

### Participants

Twenty-four participants were recruited from five teams from both in- and outpatient facilities of the department of psychotic disorders of GGZ-Drenthe in Assen, in the Netherlands. In order to be eligible to participate in this study, the participants had to meet the following criteria: (1) a diagnosis in the psychotic spectrum according to DSM-IV-TR criteria, verified by the Mini-SCAN; (2) age of 18 years or older; (3) ability to give informed consent. Exclusion criteria were as follows: (1) PANSS mean positive symptoms ≥5; (2) substance dependence (not substance abuse), verified by Mini-SCAN; (3) IQ < 70, estimated by the onsite therapist who was treating the client; (4) pregnancy; (5) co-morbid personality disorder or co-morbid neurological disorder, both verified by onsite therapist.

### Procedure

Eligible clients were initially informed about the intervention by their case managers or clinicians. Subsequently, the research team provided interested clients with more information by telephone, mail and/or through open information meetings. After two weeks clients were contacted again for their final decision. When they agreed to participate, a screening interview was planned to obtain written informed consent and to assess whether the study criteria were met. Three therapy groups of eight participants each were scheduled. Before and after BEATVIC pre and post assessments were performed.

### Intervention

BEATVIC consists of 20 weekly group sessions of 75 minutes. All sessions are led by a psychomotor therapist and an expert by experience. The intervention contains five modules each targeting specific risk factors (see Fig. 1). Every session starts with a warming-up followed by kickboxing exercises and one or two thematic (kickboxing) exercises. The first module focusses on self-stigma and is an introductory module during which participants get to know each other and are introduced to kickboxing techniques. The focus of the second module, entitled “recognizing dangerous behaviour”, lies on social cognition and participants practice identifying threatening non-verbal signals. They are stimulated to share and verify their own perception of situations and to consider other people’s perspectives. The third module focuses on insight and again on social cognition and is entitled “how others see me”: people learn to look at themselves through the eyes of others. Special attention is given to the way body posture influences the interaction both for others and for oneself. The fourth module concerns the theme “aggression regulation”, during which participants learn not only how to cope with aggression of others but also to recognize, regulate and control their own anger. The aim of this module is to adequately balance between improving resilience, while also preventing aggressive behaviour. Module five repeats and combines the themes and exercises that were important for each specific group. Each session ends with cooling-down and a discussion of the risk factors that were addressed. The latter will help people to make a connection between experiences during the therapy and daily life situations. In addition, after and during each session the participants check their arousal level and do a calming breathing exercise. Furthermore, participants are stimulated to continue kickboxing or to engage in other sports after the intervention. A group visit to a training center in the region and/or a guest lesson from a local trainer are offered to facilitate this.

### Measures

#### Screening interview

During the screening interview the DSM diagnosis and the absence of alcohol and drug addiction were verified by the *mini Schedules for Clinical Assessment in Neuropsychiatry* (miniSCAN; 2011 Dutch version) [27]. The *Positive and Negative Syndrome Scale (PANSS),* which consists of a 30 item rating scale based on a semi-structured interview*,* was administered during pre and post assessment, first to verify the absence of florid psychosis and, second as an outcome measure indicating the change in severity of the symptoms [28]. Finally, demographic variables including gender, age, family contact, living situation and daily activities were collected.

#### Feasibility of the intervention and application of an RCT

To gain knowledge about the feasibility of the intervention, the willingness of the therapists to refer participants and the willingness of the clients to participate were explored. In a logbook adherence, drop-outs and time schedules were registered. After each session and during the final evaluation, trainers and participants were asked whether they observer or experienced any adverse events at home or during a session, this was also registered in a logbook. In addition, the clinicians and case managers were asked to report possible negative side effect of the intervention in their client.

#### Evaluation and improvement of the intervention protocol

Every session was evaluated with the participants (during the group discussion) and subsequently by the psychomotor therapist, the expert by experience, the kickboxing expert and the researchers who developed the intervention. All exercises were reviewed with regard to the content (were the risk factors addressed?), suitability for the target group (e.g. mentally or physically not too demanding?), arousal levels (was stress increased or decreased?), and learning curve (how often should the exercise be repeated before the group managed the technique?). Furthermore, outcomes of the evaluation of each session were registered in a log and suggestions for improvement were discussed. In the post treatment assessment participants also completed a qualitative evaluation questionnaire including eleven open questions about the therapy and eighteen items about possible outcomes (e.g. ‘Due to the therapy: I have more self-esteem’, ‘I can prevent a fight’, rated from 1 ‘I totally disagree’ to 7 I totally agree).

#### Exploration of outcome measures

In general, the aim of a feasibility study was to explore some of the important outcome measures for the RCT, not to test all risk factors as the effect on those will be investigated in the RCT [29]. In our study two different victimization and perpetration questionnaires were explored, as well as one questionnaire on social behaviour and two on aggression regulation.

#### Victimization and perpetration

Three subscales of the *Dutch crime and victimization survey* (Integrale veiligheidsmonitor IVM [30], an adaptation of the international crime and victimization survey, were used: personal crimes, property crimes and perpetration.

For comparison, there is IVM data available on 1729 people from the general population who live in the same region as the study participants and who were interviewed at the time of this study [30]. While the IVM has been used in large surveys with people with Severe Mental Illness [31] and in studies with people with psychosis [14] no psychometric information is available. However, there are no indications of invalidity of the response in these groups. Since the examined time period is one to 5 years, the instrument was not thought to be sensitive to changes over the intervention period of 5 months. Moreover, as the incidence of crime is low, in this feasibility study no changes in victimization were expected after the intervention period. Therefore, the IVM was not included in the post measurement.

The revised *Conflict Tactics Scale (CTS2)* [32], assesses whether a respondent was involved in various types of psychological or physical conflicts and their reactions. The following subscales are distinguished: psychological aggression, physical assault, sexual coercion, physical injury and negotiation. Since victims not always see themselves as having experienced abuse, participants are asked not about attitudes, emotions and cognitive behaviours, but to indicate whether 39 forms of conflict related behaviours applied to themselves or their partner in a given time period. In our study we were interested in a broader range of social interactions and thus changed the word ‘partner’ to ‘someone’. Besides the prevalence, it is possible to calculate the frequency (or chronicity) in which an incident occurs. Frequency was categorized as once, twice, 3–5, 6–10, 11–20 or > 20 times in the previous 5 months [33]. As the CTS2 measures more subtle forms of victimization than the IVM, prevalence rates were calculated at baseline and the frequency of incidents at both pre and post measurement were used to explore possible changes. The internal consistency, reliability and construct validity of the CTS2 is good [32].

#### Social behaviour

*The Inventory of Interpersonal Situations (IIS)* measures social anxiety [34]. Respondents need to report on the frequency of occurrence and the level of discomfort they experience in 35 different social situations, ranging from 1 ‘no discomfort’ to 5 ‘very much discomfort’. Five subscales are distinguished: giving criticism, expressing opinions, giving compliments, initiating contacts, and positive self-evaluation. This questionnaire has been proven to be sensitive to change in social anxiety resulting from social interventions for people with a severe mental illness [35] and the reliability and validity are good [34]. The ISS has a Dutch norm group from the general population (*n* = 580) and the scaled scores are divided on a 7-point scale ranging from ‘very low’ to ‘very high’ [36].

#### Aggression regulation

To assess aggression regulation we used the Dutch translation of The *State Trait Anger Expression Inventory (STAXI)* [37]*.* This instrument measures to what extent participants internalize or externalize feelings of anger and assesses their control over expression and containment of these feelings of anger. Participants respond by rating 40 items on a scale ranging from 1 ‘almost never’ to 4 ‘almost always’. The STAXI has been proven to be sensitive to changes in aggression regulation resulting from a dance/movement therapy in people with schizophrenia [38], has good to high psychometric properties [39]. The STAXI has a Dutch norm group from het general population (*n* = 464) [40],

The *Novaco Anger Scale-Provocation Inventory (NAS-PI)* was added to gain insight in how people experience anger and what kind of situations provoke anger. A total score for anger disposition is calculated with 48 items divided into three domains (cognitive, arousal and behavioural). Participant rate the items on a 3-point scale ranging from 1 ‘never true’ to 3 ‘always true’. The second part is the provocation inventory, with 25 items on anger-eliciting situations to be rated on a 4-point scale ranging from 1 ‘not at all angry’ to 4 ‘very angry’. The NAS-PI has previously been used for people with a psychotic disorder [41] and has good reliability and validity [42]. The NAS-PI has a Dutch norm group of 160 male preparatory secondary vocational education students [43].

#### Possible influential risk factors

To monitor alcohol and drug use a screening list to check for the risk of substance dependence (in Dutch *Screening Risico op Verslavingsproblemen*; [44] was applied. The instrument consists of eleven questions to determine the amount of alcohol and drugs the participant uses in 1 week or month. To examine whether participants have experienced trauma and potential trauma related symptomatology the *Trauma Screening Questionnaire (TSQ)* was administered. The TSQ is a short screening instrument that contains five re-experiencing and five arousal items from the DMS-IV PTSD criteria (e.g. “upsetting dreams about the event” and “difficulty falling or staying asleep”) participants were asked to state whether they experienced these trauma related symptoms twice in the past week (yes/no). Both sensitivity and specificity of the TSQ are high [45]. The PANSS (see screening interview) was also used to measure possible influential risk factors. Video-recorded PANSS interviews were rated by independent and trained screeners, who were blind to the moment, pre or post, of assessment.

### Statistical analyses

To explore the outcome measures, pre and post treatment outcomes on each instrument were compared separately using a paired sample t-test (two sided). Alpha was set at 0.05 and no Bonferroni corrections were made due to the explorative nature of the feasibility study. We tested two sided because we wanted to explore both sides of the distribution just in case of unexpected results, for example, if kickboxing leads to more aggression instead of less aggression. In order to check the assumptions we used boxplots, QQ-plots and the Shapiro Wilk test. When assumptions were violated the Wilcoxon Signed Rank test was used. All tests were executed with the SPSS package for IBM statistics version 23.0.

As attendance varied between participants, it might be possible that some of the participants, who missed multiple sessions, obtained less information and exercise and therefore differ from high attenders. Therefore, pre-post analyses were performed twice: once including all completers and again including only the high attenders who participated in at least 75% of the sessions. The results of all completers are reported unless the description in the results says otherwise.

## Results

### Feasibility of the intervention and application of an RCT

After the therapists and case-managers received detailed information about the intervention and the feasibility study, all teams agreed to participate and were willing to refer clients. In four of the five teams the case load was screened immediately for eligible patients while one team started a month later due to shortage of staff. It took approximately two months, and 155 invitations to clients to include 24 clients. The main reasons for not participating were lack of time, not feeling the need for resilience therapy, no interest in kickboxing, or not willing to participate in the pre and post assessments. Sample characteristics are displayed in Table 1.

**Table 1:** Sample characteristics

	Completers	Drop-out
N	17	7
Age mean (SD)	35.9 (10.1)	31.0 (12.1)
Male n (%)	13 (76.5)	5 (71.4)
Living situation n (%)		
Alone	11 (64.7)	1 (14.3)
Partner	0 (0.0)	1 (14.3)
Friends	1 (5.9)	0 (0.0)
Family	2 (11.8)	0 (0.0)
Supported housing	3 (17.7)	5 (71.4)
Family contact n (%)		
1–7 times a week	14 (82.4)	5 (71.4)
1–3 times a month	3 (17.7)	2 (28.6)
Daily activity n (%)		
Part-time paid job	2 (11.8)	0 (0.0)
Student	1 (5.9)	1 (14.3)
Volunteer or other activities	8 (47.1)	2 (28.6)
Unemployed	6 (35.3)	4 (57.1)
Diagnosis n (%)		
Paranoid schizophrenia	7 (41.2)	0 (0.0)
Disorganized schizophrenia	0 (0.0)	3 (42.9)
Depression with psychotic features	1 (5.9)	0 (0.0)
Schizophreniform disorder	4 (23.5)	0 (0.0)
Delusion disorder	1 (5.9)	1 (14.3)
Brief psychotic disorder	1 (5.9)	2 (28.6)
Psychotic disorder NOS	3 (17.7)	1 (14.3)

**Fig. 2:**
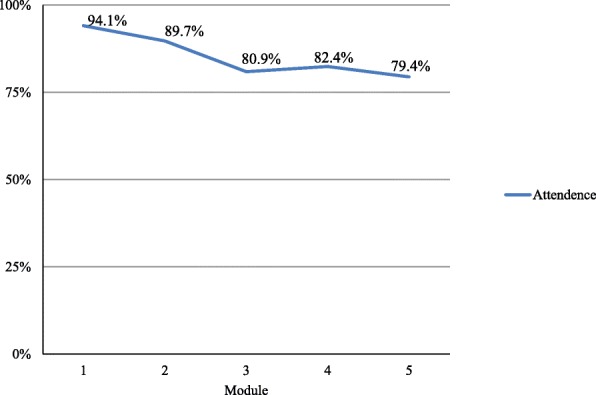
Percentage attendance per module

During the intervention, seven participants dropped out: three persons never attended a session, three participants attended only one session, and one participant dropped out after four sessions. There were multiple reasons for dropout such as a lack of motivation, lack of time or physical or mental problems. Due to the small sample size we did not tested differences between characteristics of this dropout group and the completers statistically. However, compared to the completers, the dropout group consisted of relatively more young people, and more people living in supported housing facilities. Three out of seven dropouts were diagnosed with disorganized schizophrenia versus none in the group of completers (see Table 1). Dropouts and completers were comparable with regard to gender, alcohol and drug use, symptoms score of the PANSS, amount of family contact, victimization, trauma, social behaviour, and aggression regulation. The mean attendance was 85.3% (SD = 13.4, range 50–100%), and 88% of the participants completed 75% (fifteen sessions) or more of the twenty sessions. Attendance was highest during the first two modules and lowest during modules 3, 4 and 5 (see Fig. 2). Attendance was especially affected when the continuity of the sessions was interrupted due to holidays. In these cases participants reported to forgot to show up. Other reasons were no time, no transportation, mental problems or other obstacles like the flu or lack of motivation. No adverse advents considered to be related to the intervention were reported.

### Evaluation of the intervention protocol

Of the seventeen participants who completed the evaluation form, ten persons indicated that 20 weekly sessions were sufficient, while five of them recommended more sessions (between 25 and 40 sessions), and two individuals preferred a more intense course of therapy with two sessions per week. Fourteen participants reported that the 75 min now set for each session was appropriate, two suggested longer sessions, and one thought 75 min was too long. Overall, participants enjoyed the therapy and thought it was helpful and informative. The sequence order and structure of the modules were positively evaluated and the (thematic) exercises within each session were rated as relevant.

The kickboxing exercises were reported to be doable for all participants, regardless of weight, strength, stamina or flexibility. Within-group differences with regards to strength or stamina were not a problem; everyone found themselves participating at their own level with exercises adapted in case of physical problems. Table 2 shows the outcomes of the qualitative evaluation questionnaire. According to the participants the intervention especially had a positive effect on identifying and setting boundaries, recognizing those of others, self-esteem, faith in own strength, confidence, recognizing dangerous situations, feelings of safety, and people though they had a lower change of becoming a victim. Most mean scores increased when only the high attenders, who attended 75% or more of the sessions, were included in the analysis.

**Table 2:** Outcomes qualitative evaluation questionnaire

Due to the therapy	Completers Mean (SD) *N* = 17	High attenders Mean (SD) N = 13^a^	Due to the therapy	Completers Mean (SD) N = 17	High attenders Mean (SD) N = 13^a^
I enjoy social contacts more	4.59 (0.80)	4.54 (1.04)	I experience less self-stigma	4.47 (1.59)	5.00 (1.00)
I have more social contacts (outside therapy)	4.18 (1.33)	4.31 (0.63)	I have more self-esteem	5.24 (1.56)	5.46 (1.27)
I recognize other people’s boundaries better	5.29 (0.85)	5.38 (0.87)	I am more assertive	4.76 (1.35)	5.08 (0.95)
I can identify my own boundaries better	5.59 (1.06)	5.77 (0.93)	I have more faith in my own strength	5.47 (1.18)	5.46 (1.05)
I can set my own boundaries more easily	5.35 (1.06)	5.54 (0.88)	I have more confidence	5.44 (0.96)	5.42 (1.08)
I recognize dangerous situations better	5.18 (0.95)	5.23 (0.60)	I feel safer on the street	5.35 (1.00)	5.38 (1.04)
I can prevent a fight	4.76 (0.97)	4.77 (0.83)	I have more respect for others	4.81 (0.83)	4.67 (0.78)
I recognize when I become angry or agitated	4.35 (1.37)	4.69 (0.86)	Others have more respect for me	4.63 (0.81)	4.42 (0.67)
I have more control over my emotions	4.53 (1.01)	4.62 (0.87)	I am less likely to become a victim	5.35 (1.00)	5.54 (0.97)

^a^Attended to 75% or more of the sessions; Scoring range: 1 totally disagree, 2 disagree, 3 somewhat disagree, 4 neutral, 5 somewhat agree, 6, agree, 7 totally agree

Although it was not a goal of the intervention, some of the participants did notice that they had lost weight, improved their stamina and endurance, and were drinking less alcohol at the end of the intervention. None of the participants reported alarming arousal levels during or at the end of a session. Several participants noticed that their arousal level was lower after a session and that they felt more relaxed.x

### Improvement of the intervention protocol

Based on the information gathered by means of the evaluation questionnaire and feedback from participants, trainers, expert by experience, kickboxing expert and researchers, several adaptations in the intervention protocol for the RCT were made after this pilot. First of all, it was noticed that in general more time than expected was needed for the participants to fully understand a theme, manage a technique or to make a kickboxing combination routine. For this reason multiple repetitions of important themes and techniques were added to the protocol, in combination with the advice to explain and practice complex kickboxing combinations in small steps. Secondly, more challenging exercises (e.g. high kick, sparring) were included in the protocol as the participants liked the challenge and it created theme-related learning opportunities. Thirdly, an intensive work-out on kickboxing pads was added to every session because participants emphasized that they enjoyed such an intensive exercise because this in particular provided positive experiences of strength and acquired kickboxing skills. Finally, although BEATVIC is a body-oriented therapy, participants positively evaluated the opportunity to talk and reflect on the therapy in the end of the session. For this reason, time was reserved for discussion at the end of each session. After the therapy ended, nine out of seventeen participants continued kickboxing at a local gym. One year later six participants still attended weekly training sessions.

### Exploration of outcome measures

#### Victimization

Table 3 shows that based on the IVM, at baseline 75% of the participants had been a victim of at least one crime in the previous five years. Both, personal and property crimes were reported by 58% of the participants. Compared to the five year rate, with 21%, the one-year victimization prevalence was approximately between three times lower, and sexual harassment or assault were not reported at all. Prevalence of victimization in the general population living in the same region was half of that in participants with all events taken into account, and only 25% in case of personal crime.

**Table 3:** Number, percentage and chronicity of victimization and perpetration

	Participants *N* = 24		General population *N* = 1729			
*IVM*	Previous year % (n)^a^	Previous five years % (n)^a^	Previous year % (n)^a^			
Property crime^b^	12.5 (3)	58.3 (14)	8.6 (149)^c^			
Attempted burglary	4.2 (1)	16.7 (4)				
Burglary	4.2 (1)	25.0 (6)				
Bicycle theft	8.3 (2)	20.8 (5)				
Theft (other)	4.2 (1)	12.5 (3)				
Vandalism	4.2 (1)	25.0 (6)	3.6 (62)			
Pick-pocketing	0.0 (0)	4.2 (1)				
Robbery	0.0 (0)	8.3 (2)				
Personal crime^d^	8.3 (2)	58.3 (14)	1.9 (33)			
Sexual harassment or assault	0.0 (0)	8.3 (2)				
Threats of violence	8.3 (2)	41.7 (10)				
2003Physical assault	4.2 (1)	16.7 (4)				
Other victimization incidents	12.5 (3)	12.5 (3)				
Total victimization^e^	20.8 (5)	75.0 (18)	12.5 (216)			
Perpetration^f^	16.7 (4)					
*CTS2* Towards participant (victimization)	Completers N = 17					
	Previous five months % (n)^a^	Pre Mdn (IQR)^h^	Post Mdn (IQR)^h^	Z	r	p
Psychological aggression^g^	47.1 (8)	0.00 (2.00)	2.00 (2.00)	− 1.98	0.48*	0.048
Physical assault^g^	29.4 (5)	0.00 (1.00)	0.00 (1.00)	−0.85	0.21	0.40
Sexual coercion^g^	0.0 (0)	0.00 (0.00)	0.00 (0.00)	−1.00	0.24	0.32
Physical injury^g^	0.0 (0)	0.00 (0.00)	0.00 (0.00)	−1.34	0.33	0.18
		Pre Mean (SD)	Post Mean (SD)	Paired Diff. (95% CI)	t	p
Negotiation^i^	94.1 (16)	6.94 (6.04)	6.69 (3.81)	0.06 (−2.44–2.56)	0.05	0.96
*CTS2* Towards someone (perpetration**)**		Pre Mdn (IQR)^h^	Post Mdn (IQR)^h^	Z	t	p
Psychological aggression^g^	41.2 (7)	0.00 (2.00)	1.00 (3.00)	0.92	0.22	0.36
Physical assault^g^	4 (23.5)	0.00 (1.00)	0.00 (0.50)	−0.17	0.04	0.86
Sexual coercion^g^	0.0 (0)	0.00 (0.00)	0.00 (0.00)	−1.00	0.24	0.32
Physical injury^g^	11.7 (2)	0.00 (0.00)	0.00 (0.00)	−0.97	0.24	0.33
		Pre Mean (SD)	Post Mean (SD)	Paired Diff. (95% CI)	t	p
Negotiation^i^	100.0 (17)	2.76 (1.56)	7.65 (4.40)	−4.88 (−6.91- -2.85)	−5.10	< 0.01

^a^ At least one incident *n* > 0; ^b^Consists of burglary, attempted burglary, bicycle theft, theft (other), vandalism, pick-pocketing, robbery; ^c^Consists of property crime without vandalism; ^d^ Consists of sexual harassment or assault, threats of violence, physical assault. ^e^ Consists of property crime, personal crime and other victimization incidents; ^f^ Consists of threats of violence, physical assault, sexual assault or other crimes (only previous year was examined); ^g^ Wilcoxon Signed Rank test; ^h^ Frequency; ^i^ Paired sample t-test. IVM = Dutch crime and victimization survey; CTS2: revised Conflicts Tactics Scale

Baseline measures of the CTS2 showed that 24% of the participants had experienced physical assault in the preceding five months. Psychological aggression was reported by 47% of the participants with no one reporting sexual coercion or physical injury. Pre and post measures revealed that the experienced frequency (or chronicity) of psychological aggression towards the participants had increased after the intervention (p 0.048). No such changes were found for the other victimization subscales.

On the negotiation items of the CTS2 only one participant reported negatively. After the intervention, the frequency of negotiation during conflict had increased (*p* < 0.01) compared to baseline.

#### Perpetration

Seventeen percent of the participants indicated that they had been the perpetrator of a crime themselves in the previous year (IVM), measured at baseline. The CTS2 results showed that 41% had used psychological aggression, 24% had used physical assault and two participants (12%) had physically injured someone in the preceding five months. None of the participants reported to have used sexual coercion. No differences between pre and post measurements were found on perpetration scores (see Table 3).

#### Aggression regulation

Compared to a Dutch norm group from the general population, participants scored one decile higher on ‘internal anger’ (mean 22.5, sd 7.0) scale and two deciles lower on ‘external anger’ (mean 21.2, sd 5.6) on the STAXI at baseline. ‘Control of internal anger’ was as high in participants as in the norm group (mean 26.0, sd 6.8) and ‘control of external anger’ was two deciles higher (mean 27.4 sd 6.4). At post measurement the mean score on control of internal anger was one decile higher than at baseline but this increase was not significant (p 0.071). The three other subscales did not show a significant change over time (see Table 4).

**Table 4:** Pre and post treatment aggression regulation and social behaviour scores

	Pre Mean (SD)	Post Mean (SD)	Paired Diff. (95% CI)	*t*	*p*
*STAXI*^*a*^ *N* = 17					
Internalizing anger	24.94 (6.69)	24.65 (6.86)	0.29 (−1.77–2.36)	0.30	0.77
Externalizing anger	17.00 (4.46)	18.24 (4.19)	−1.24 (−2.84–0.37)	−1.64	0.12
Control of internalizing	27.53 (7.75)	29.53 (4.46)	−2.00 (−4.20–0.20)	−1.93	0.071
Control of externalizing	30.35 (5.99)	30.29 (4.67)	0.06 (−2.12–2.24)	0.06	0.96
*NAS-PI*^*a*^ N = 13*					
Cognition	31.00 (3.34)	29.85 (3.53)	1.15 (−0.32–2.63)	1.70	0.11
Arousal	29.62 (3.82)	28.508 (3.93)	1.54 (0.15–2.92)	2.42	0.033
Behaviour	23.85 (4.18)	23.15 (3.29)	0.69 (−1.51–2.89)	0.69	0.51
NAS total	84.46 (10.18)	81.08 (9.74)	3.38 (−0.43–7.19)	1.93	0.077
PI total	55.90 (10.68)	54.62 (9.91)	1.31 (−2.30–4.91)	0.79	0.45
*IIS*^*b*^ N = 17	Pre Mdn (IQR)	Post Mdn (IQR)	Z	*r*	*p*
Discomfort					
Giving Criticism	21.00 (5.00)	19.00 (6.00)	−1.80	0.44	0.072
Expressing Opinions	14.00 (6.00)	14.00 (4.00)	−0.86	0.21	0.39
Giving Compliments	6.00 (3.00)	5.00 (3.00)	−1.03	0.25	0.30
Initiating contacts	11.50 (7.00)	11.00 (7.00)	−0.54	0.13	0.59
Positive self-evaluation	8.00 (3.00)	8.00 (2.50)	−0.56	0.14	0.58
Total Discomfort	77.00 (24.00)	75.00 (11.00)	−1.04	0.25	0.30
Frequency					
Giving Criticism	17.00 (4.00)	16.00 (4.50)	−0.26	0.06	0.80
Expressing Opinions	17.00 (5.00)	16.00 (2.50)	−1.67	0.41	0.09
Giving Compliments	16.00 (4.50)	15.00 (4.00)	−0.23	0.06	0.81
Initiating contacts	14.00 (6.50)	17.00 (5.50)	−0.61	0.15	0.54
Positive self-evaluation	12.00 (6.00)	13.00 (4.50)	−0.38	0.09	0.70
Total Frequency	104.00 (30.25)	101.00 (26.00)	−0.02	0.01	0.98

^a^Paired sample t-test; ^b^ Wilcoxon Signed Rank test; * high attenders who attended 75% or more of the sessions; *STAXI* State Trait Anger Expression Inventory, *NAS-PI* Novaco Anger Scale-Provocation Inventory, *IIS* Inventory of Interpersonal Situations

At pre and post measurement the participants scored both one decile lower on the NAS total score compared to the norm group (mean 89.7, sd 14.2). In accordance no significant difference was found between pre and post scores for the NAS total score as well as for the PI score. However, when only the high attenders were included in the analyses the ‘arousal’ subscale of the NAS-PI showed a significant decrease over time (p 0.033) (see Table 4).

#### Social behaviour

At baseline, the median score of the participants was ‘above average’ on the ISS compared to the norm group on the ‘total social discomfort’ scale. After therapy this decreased to ‘average’ discomforts however this change was statistically nonsignificant. At baseline the median frequency of ‘total social contacts’ scale was ‘below average’ compared to the norm group. At post measurement the median frequency of the ‘total social contacts’ scale was still ‘below average’ but again nonsignificant (see Table 4).

#### Possible influential risk factors

No differences between pre and post measurement were found on all scales of the PANSS, or on the screening risk of substance dependence questionnaire. Most participants did not experience symptoms of trauma at pre or post measurements (see Table 5).

**Table 5:** Pre and post PANSS, substance abuse and TSQ scores

N = 17	Pre Mdn (*IQR*)	Post Mdn (*IQR*)	Z	*r*	*p*
*PANSS* ^*a*^					
Positive symptoms	11.00 (4.50)	11.00 (5.00)	−0.64	0.16	0.53
Negative symptoms	10.00 (5.00)	10.00 (3.50)	−0.27	0.07	0.90
General symptoms	24.00 (9.00)	25.00 (9.00)	−0.33	0.08	0.74
Total score	44.00 (19.00)	45.00 (17.50)	−0.57	0.14	0.60
*Substance abuse* ^*a*^	20.00 (7.00)	19.00 (10.50)	−0.15	0.04	0.88
*TSQ* ^*a*^	0.00 (2.00)	0.00 (3.00)	−0.34	0.08	0.73

^a^Wilcoxon Signed Rank test; *PANSS* Positive and Negative Syndrome Scale, *TSQ* Trauma Screening Questionnaire

## Discussion

To our knowledge, BEATVIC is the first body-oriented resilience therapy that aims to decrease victimization risk in people with a psychotic disorder. The goal of this study was to evaluate its feasibility in order to evaluate the usefulness of a larger RCT that can shed light on efficacy of BEATVIC.

### Feasibility of the intervention and application of an RCT

Our findings support the feasibility of BEATVIC. The mental health professionals were willing to refer to BEATVIC and a relatively large group of clients (one out of every six invited) was willing to participate. The mean age of the participants was 36 years. The oldest included participant was 51 years old, which indicates that BEATVIC appeals to a wide variety of people.

The dropout rate of 29% was as could be expected based on previous studies: the estimated dropout rate of physical activity interventions for people with schizophrenia lies between the 20 and 35% [46]. Six out of seven dropouts attended none or only one session. It is possible that, despite all the provided information, these participants were not fully aware beforehand of what the treatment would entail and how much time would be involved. To prevent dropout it is recommended to verify whether the client received and understood all the information.

Overall attendance was good compared to other interventions [47, 48]. This finding is particularly relevant as high attendance is important because of the intensity of BEATVIC and its hierarchical structure where the kickboxing exercises are concerned. Non-attendance of two or more sessions means that important exercises are missed and participants fall behind in the group. In accordance, the high attenders who were present at more than 75% of the sessions reported that they had improved more on the addressed risk factors, compared to the low attenders. This is in line with a study of Scheewe et al. [49] who only found significant improvements in people who attended more than 50% of the exercise sessions. In line with these experiences, it was decided that to measure effectiveness in the RCT, we will not only use an intention-to-treat analysis but also perform a per-protocol analysis.

### Evaluation and improvement of the intervention protocol

The BEATVIC therapy was positively evaluated by the trainers and the participants. Overall, the number, duration and sequence order of the sessions were seen as adequate, and the (thematic) exercises were rated as relevant. In the results section, an overview of implemented improvements was presented regarding the number of repetitions, the right amount of challenge and intensity of exercises, and total discussion time. Participants enjoyed the exercises and they subjectively reported positive effects on several factors.

Some of the participants noted that they had lost weight and felt that their stamina and endurance was improved. To objectively measure this, we will include physical outcomes in the RCT as this is particular relevant for the target group who also faces increased metabolic risks [50, 51]. This study has shown that it is appropriate to use kickboxing in a body-oriented therapy. The exercises were at a feasible level for all participants and people enjoyed learning the techniques which was confirmed by the fact that half of the group continued kickboxing at a local gym.

### Exploration of suitable outcome measures

To find suitable instruments for the RCT we explored some of the important outcome measures.

#### Victimization and perpetration

The IVM and the CTS2 showed to be adequate instruments to detect victimization incidents. Although there is some overlap in subscales, both can be used complementary because of their specific characteristics. With the IVM the victimization prevalence can be compared to the general population who live in the same neighbourhood while the instrument also shows international comparability [52]. The IVM also provides information on victimization both in the preceding year (in our case 21%) and the preceding 5 year (75%). Subsequently, some types of victimization (e.g. sexual assault, robbery) were only reported during the 5 year period and not during the 1 year period. This indicates the importance for a follow-up in the RCT. Preferably more than 1 year to capture the less frequent victimization types.

The CTS2 measures more subtle forms of victimization and takes into account the frequency in which an incident occurs. In our study more people reported physical assault on the CTS2 (29%) than on the IVM (4.2%). A possible explanation might be that the CTS2 asks more specific assault questions which may elicit higher recall of incidents. The CTS2 showed to be sensitive to change: more psychological aggression was reported after the intervention than before and participants more often used negotiation as a communication technique.

#### Aggression regulation

The NAS-PI and the STAXI were used to explore whether these tests could capture changes in aggression regulation induced by the intervention. Only a significant improvement on the arousal subscale of the NAS-PI for the high attenders, but no other significant changes were found. At baseline on average the STAXI and NAS-PI scores did not indicate that the participants had aggression regulations problems and there may not have been much room for improvement. In the future it is recommended to perform a subgroup analysis for participants who have aggression regulation problems at the start of the treatment.

#### Social functioning

In this study the IIS was used to explore whether this test could capture changes in interpersonal situations. No significant changes were found and therefore we decided to use another test for the RCT. Besides a lack of power due to the small sample size, it is possible that the participants did not significantly improve on the IIS because it measures a broad spectrum of interpersonal situations. It is expected that the intervention can improve social functioning, as other studies that included martial arts found positive results on social behaviour [53, 54]. In the future it is recommended to measure aspects and/or underlying mechanisms of social functioning that are related to victimization, for example assertiveness and impaired social cognition.

#### Limitations of the study

First of all, because no control group was included no conclusions can be formulated as to whether the (subjective) improvements derive from the group meetings and time with the trainers or from BEATVIC. Secondly, since not all participants had been victimized at baseline, it was difficult to find improvements in this respect. These participants may have been appealed by the kickboxing-element of the therapy, rather than working on their resilience.

## Conclusion

In this feasibility study BEATVIC was found to be a feasible intervention for people with a psychotic disorder. Both mental health professionals and clients gave positive evaluations and attendance was good. Trainers, participants and scientists gave suggestions for small improvements in the intervention protocol. Our results support the evaluation of BEATVIC in a RCT.

## Abbreviations

CTS2: Conflict tactics scale
IIS: Inventory of interpersonal situations
IVM: Integrale Veiligheidsmonitor (Dutch crime and victimization survey)
NAS-PI: Novaco anger scale-provocation inventory
PANSS: Positive and negative syndrome scale
PMT: Psychomotor therapy
STAXI: State trait anger expression inventory
TSQ: Trauma screening questionnaire
